# The roles of E3 ubiquitin ligases in cancer progression and targeted therapy

**DOI:** 10.1002/ctm2.1204

**Published:** 2023-03-07

**Authors:** Chibuzo Sampson, Qiuping Wang, Wuxiyar Otkur, Haifeng Zhao, Yun Lu, Xiaolong Liu, Hai‐long Piao

**Affiliations:** ^1^ CAS Key Laboratory of Separation Science for Analytical Chemistry Dalian Institute of Chemical Physics Chinese Academy of Sciences Dalian China; ^2^ University of Chinese Academy of Sciences Beijing China; ^3^ Department of Orthopedics Dalian Second People's Hospital Dalian China; ^4^ Department of Stomatology Dalian Medical University Dalian China

**Keywords:** cancer, E3 ubiquitin ligases, targeted therapy, ubiquitination

## Abstract

Ubiquitination is one of the most important post‐translational modifications which plays a significant role in conserving the homeostasis of cellular proteins. In the ubiquitination process, ubiquitin is conjugated to target protein substrates for degradation, translocation or activation, dysregulation of which is linked to several diseases including various types of cancers. E3 ubiquitin ligases are regarded as the most influential ubiquitin enzyme owing to their ability to select, bind and recruit target substrates for ubiquitination. In particular, E3 ligases are pivotal in the cancer hallmarks pathways where they serve as tumour promoters or suppressors. The specificity of E3 ligases coupled with their implication in cancer hallmarks engendered the development of compounds that specifically target E3 ligases for cancer therapy. In this review, we highlight the role of E3 ligases in cancer hallmarks such as sustained proliferation via cell cycle progression, immune evasion and tumour promoting inflammation, and in the evasion of apoptosis. In addition, we summarise the application and the role of small compounds that target E3 ligases for cancer treatment along with the significance of targeting E3 ligases as potential cancer therapy.

## Graphical abstract

E3 ubiquitin ligases are important players in cellular processes by ubiquitinating substrate proteins in disease progression such as cancer. Owing to this, scientists attempt to identify safe and bioavailable compounds targeting E3 ligases. In this review, we summarise the roles of E3 ligases in cancer‐related pathways, highlight the application and clinical significance of small molecule inhibitors and discuss further directions

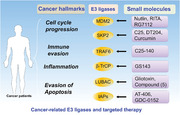

## 1 INTRODUCTION

Cancer is a heterogeneity disease characterised by diverse dysregulated biological processes.^1^ Normal cell growth is controlled by extracellular and intracellular cues that direct the determination of various cellular processes such as cell signalling, cell cycle, DNA repair, transcriptional regulation and apoptosis.^2^ The regulation of these cellular processes is greatly influenced by post‐translational modifications. In principle, these are achieved by regulating the activity of proteins involved in the various cellular processes with the dysregulated post‐translational modification associated with aberrant cell function and the manifestation of diseases such as cancer.^3^, ^4^ Ubiquitination is one of the most important post‐translational modification that involves the transfer of ubiquitin, a small regulatory protein, to specific substrates for proteasome degradation.^5^ Non‐proteolytic functions of ubiquitin have also been reported including transcription and translation regulation, DNA repair, protein trafficking, signalling activation and suppression.^6^, ^7^, ^8^

Ubiquitin‐substrate linkage is an isopeptide bond between the C‐terminal (glycine 76) of ubiquitin and ɛ‐amino group of the lysine residue of substrate protein.^9^ Ubiquitin can also be conjugate to threonine, serine or cysteine residue as well as the free N‐terminal residue (α‐NH_2_ group) of the substrate.^10^, ^11^, ^12^ Substrates are monoubiquitinated, multi‐monoubiquitinated or polyubiquitinated. Monoubiquitination is mostly implicated in non‐proteolytic function as exemplified in histone monoubiquitination implicated in chromatin modification, DNA damage response and repair signalling.^13^ Histone 2A (H2A) ubiquitination at lysine 119 (K119) by polycomb repressor complex 1 (PRC1) and histone 2B (H2B) ubiquitination at lysine 120 (K120) by RING finger complexes (RNF20/40) are the well‐known histone monoubiquitination whose deregulation has been identified in several malignancies.^13^, ^14^ In polyubiquitination, the first ubiquitin binds the specific lysine residue of the protein substrate while the incoming ubiquitin binds the internal lysine of the previously bound ubiquitin forming Ub–Ub chain. There are seven lysine residues and an N terminal methionine (K6, K11, K27, K29, K33, K48, K63 and M) within ubiquitin moiety for polyubiquitination chain assembly.^15^ Polyubiquitin chains via K48 and K11 are known to target substrate for degradation by downstream 26S proteasome.^16^, ^17^ K63‐ and M1‐linked linear ubiquitin chains perform non‐proteolytic functions and are critical players in immune response and inflammatory signalling.^17^, ^18^, ^19^ Other ubiquitin linkages are not fully characterised although they have been shown to function in immune response, DNA damage and repair response, mitophagy and cellular stress response.^20^, ^21^, ^22^

Three component enzymes are engaged in the ubiquitination process including ubiquitin‐activating enzyme (E1), ubiquitin‐conjugating enzyme (E2) and ubiquitin ligase (E3).^23^ The specificity of the ubiquitin system is determined by E3 ligases which specifically select proteins for ubiquitination.^24^ E3 ligases can select target proteins by identifying a specific peptide motif termed degron in the substrate.^25^ Such degrons include N‐terminal and C‐terminal degrons,^26^ proline‐rich motif (PPRX) typically recognised by WW domain containing E3 ligase,^27^ phospho‐degron common for WD40 containing E3 ligase^28^ and D box and Ken box found commonly in APC/C substrates.^29^ Plethora of studies have shown that E3 ligases are an important player in cancer progression explainable by their regulatory effect on proteins involved in various cellular processes. Owing to this fact, there has been a growing attempt to identify safe and bioavailable compounds that target E3 ligases with high specificity for cancer therapy. Herein, we summarise the roles of E3 ligases in cancer promoting pathways like cell cycle progression, immune evasion, inflammatory signalling and apoptosis escape. In addition, we highlight the application and clinical significance of small molecule inhibitors of E3 ligase as well as small molecule degraders, recruiting E3 ligase for target protein degradation.

## 2 CLASSIFICATION OF E3 UBIQUITIN LIGASES

There are over 600 E3 ligases identified hitherto, each targeting specific substrate proteins. All E3 ligases can be grouped into three families; the really interesting new gene (RING) finger family, the homologous to E6AP C terminus (HECT) family and the RING between RING (RBR) family.

### 2.1 RING finger family E3 ligases

The RING finger E3 ligases make up the largest E3 family that contains the RING or U‐box catalytic domain. The canonical RING finger is a cysteine‐rich domain with the sequence of Cys‐X_2_‐Cys‐X_(9‐39)_‐Cys‐X_(1‐3)_‐His‐X_(2‐3)_‐Cys‐X_2_‐Cys‐X_(4‐48)_‐Cys‐X_2_‐Cys (where X represents any amino acid). It uses the cysteine and histidine residues to coordinate two zinc ions in an eight ligands cross based structure.^30^, ^31^ RING finger E3 ligases do not bind ubiquitin directly but mediate the transfer of ubiquitin from bound E2 (E2‐Ub) to the target substrate.^32^ Ring finger E3 ligases may function independent of any auxiliary protein, as a monomer or homodimer, such as TRIM (Tripartite Motif containing E3 ligases), TRAF6 (TNF Receptor Associated Factors 6), cIAP (cellular Inhibitor of Apoptosis), XIAP (X‐linked inhibitor of apoptosis), RING finger (RNF) containing proteins and MDM2 (Murine Double Minute 2)^33^, ^34^, ^35^, ^36^, ^37^ or associate with another protein and function as a heterodimer or multisubunit complex. Heterodimeric ring finger E3 ligases include TRAF heterodimers,^38^ MDM2/MDMX,^39^ BRCA1/BARD1^40^ and RING1B/BMI‐1.^41^ MDMX, BARD1 and BMI‐1 in the above heterodimers have ring domain, but do not possess E3 ligase activity therefore they associate with the E3 ligase ring domain counterpart to promote substrate ubiquitination.

Multisubunit ring finger E3 ligases include the Cullin Ring Finger ligases (CRL) and Anaphase Promoting Complex/cyclosome (APC/C). The CRL is a large family, containing Cullin scaffold protein, E2 binding RING‐box protein (Rbx1 and Rbx2), adaptor protein and substrate recognition protein.^42^ The substrate recognition proteins bind adaptors while the Cullin forms a central scaffold that bridges Rbx1/2 and the adaptor‐substrate recognition protein. Differences in Cullin type (Cul1, Cul2, Cul3, Cul4A, Cul4B, Cul5, Cul7 and Cul9) form the basis for different groups of the CRL subfamily.^43^ The specificity of substrate recruitment is determined by substrate recognition proteins with over 400 kinds identified.^44^ SCF (**S**KP1‐**C**ullin1‐**F**‐box) E3 ligase is the largest CRL family with SKP1 as the adaptor and F‐box proteins as the substrate recognition unit.^45^ There are 69 F‐box proteins encoded in human genome which can be grouped into three subfamilies based on their substrate recruiting domain: F‐box with WD‐40 domain (FBXW), F‐box with leucine rich repeat (FBXL) and F‐box with other domains (FBXO).^46^, ^47^ F‐box proteins are important regulators of diverse cell functions; an example is SKP2 (also called FBXL1), a popular oncoprotein that mediates the degradation of CDK inhibitors to enable G1 to S phase cell cycle progression.^48^ The other ring finger complex, APC/C, functions in cell cycle mitotic progression. They contain 19 subunits that are grouped into three sub‐complexes: scaffolding platform, ring finger containing catalytic core and the tetratricopeptide repeat (TPR).^49^ APC/C requires co‐activator proteins, CDC20 and CDH1 for substrate recruitment to the APC/C complex. The co‐activator enters the APC/C complex by binding APC3 and APC8 in the TPR sub‐complex,^50^, ^51^ recognises and recruits substrate containing KEN‐box or D‐box to the APC/C complex for ubiquitination.^29^

### 2.2 HECT family E3 ligases

The HECT families of E3 ligase bear HECT catalytic domain at their Carboxyl terminal lobe. A flexible link with a cysteine active site connects the N terminal and C terminal lobes.^52^ This cysteine active site, confers on them a distinct mechanism of ubiquitination from the ring finger families. In HECT‐mediated ubiquitination, ubiquitin from E2 first forms an intermediate thioester bond with the cysteine before being transferred to the target substrate.^53^ Twenty‐eight HECT E3 ligases have been identified in human and are grouped into three different subfamilies based on N terminal domain including the NEDD4 family containing N‐terminal WW and C2 domain, the HERC family containing N‐terminal RCC‐like domain (RLD) and other HECT with variable N‐terminal domain. HECT members are actively involved in several cellular processes that drive cancer progression. For example, E6AP, a member of other HECT family drives cervical cancer by associating with E6 protein of human papillomavirus to promote proteasome degradation of p53^54^, ^55^; the NEDD4 family members WWP1 and NEDD4 promote PI3K/AKT signalling by catalysing the ubiquitin‐mediated degradation of PTEN^56^; ITCH, another NEDD4 member, is an important regulator of immune response.^57^, ^58^

### 2.3 RBR family E3 ligases

The RBR ligases are considered the smallest E3 family with only 14 members among which some well‐known E3 ligases such as Parkin, HHARI, TRIAD1, HOIP, HOIL‐IL and RNF144 are included.^59^, ^60^ Canonical RBRs consist of three components: RING1 with an E2 binding domain, RING2 with a catalytic cysteine residue and an In‐between Ring domain (IBR).^61^ RBRs are described as RING–HECT hybrid because the RING1 is structurally similar to Ring type E3 ligase, while the RING2 bears an exposed catalytic cysteine residue that forms intermediate thioester linkage with ubiquitin during substrate ubiquitination just like the HECT family.^62^ Traditional RBRs exist in the form of N‐RING1–IBR–RING2‐C; however, they can contain additional domains at the N terminal, middle or C terminal. These outside domains confer a characteristic auto‐inhibitory action on RBR, for example, the C‐terminal Ariadne domain of HHARI blocks RING2 active site leading to HHARI autoinhibition.^60^ Parkin, a tumour suppressor RBR implicated in neurodegeneration disease and innate immune response, is kept in an autoinhibition state by three outside domains, ubiquitin like domain (Ubl), Ring0 domain and a REP domain.^63^, ^64^ REP and Ubl mask E2 binding site on RING1, while Ring0 binds to RING2 and blocks its catalytic cysteine residue thereby keeping Parkin in an autoinhibition state.^65^, ^66^ Phosphorylation of the Ubl domain by PTEN‐induced kinase 1 (PINK1) and the binding of phosphorylated ubiquitin to Parkin disrupt the autoinhibition state, releasing Parkin for activity.^67^, ^68^ LUBAC (Linear ubiquitin chain assembly complex), another RBR ubiquitin ligase active in innate immune response, is composed of HOIP, HOIL‐IL and SHARPIN that generates linear polyubiquitin chains.^19^, ^60^ HOIP bears ubiquitination catalytic activity but is kept in the inactive auto‐inhibited state by the ubiquitin‐associated (UBA) domain at its N terminus. To distort the autoinhibition, the ubiquitin like domain of either HOIL‐IL or SHARPIN interacts and forms a complex with the UBA domain of HOIP thereby activating HOIP for M1 linear ubiquitination.^69^, ^70^ In addition to the established E3 ligase catalytic activity of HOIP, HOIL‐1L has been identified as E3 ligase that catalyses oxyester linked self‐monoubiquitination and monoubiquitination of protein components of Toll‐like receptor (TLR).^12^

## 3 ROLES OF E3 LIGASES IN CANCER HALLMARKS

Hanahan and Weinberg^71^ described ten hallmarks of cancer which are acquired strategies by cancer cells that enable their survival, growth and metastasis. Significant numbers of evidences have demonstrated the participation of ubiquitination in cancer hallmark pathways. For brevity, we will particularly summarise recent findings on the implication of E3 ubiquitin ligases in cell cycle progression, immune evasion and inflammation and the evasion of apoptosis.

### 3.1 Role of E3 ubiquitin ligases in cell cycle progression

**FIGURE 1:**
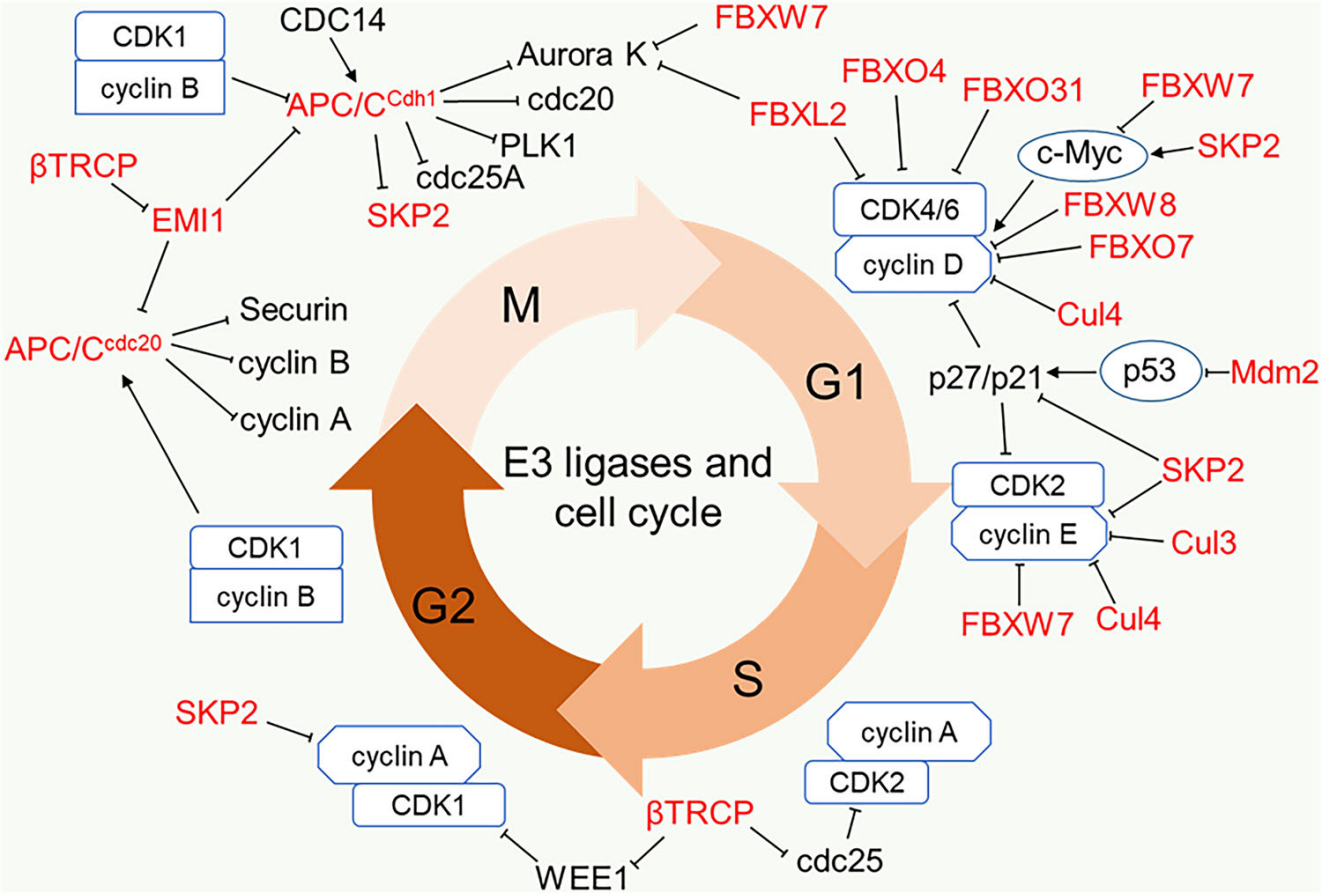
E3 ligases in cell cycle regulation. E3 ligases regulate cell cycle progression via regulating the CDK and cyclin activity at the four phases of the cell cycle. E3 ligases majorly act directly on Cyclins and mediate their ubiquitination and degradation, but for CDKs, E3 ligases act on the primary CDK regulators such as CDK inhibitors (p27, p21, p57), and mediate their ubiquitination and degradation.

Cancer cells abrogate cell cycle regulation to sustained proliferation and progression. The cell cycle is a series of incidents that progress through four phases: gap1 phase (G1 phase), DNA synthesis phase (S phase), gap2 phase (G2 phase) and mitosis phase (M phase). The cyclin‐dependent kinases (CDKs) are key regulators of the cell cycle that drive cell division by forming complex with cyclins with distinct CDK/cyclin complex operating at different phases.^72^, ^73^ Cyclins are short‐lived proteins degraded during cell cycle by E3 ligases,^74^ while CDKs are relatively stable; however, their activities can be inhibited by other cell cycle regulatory proteins among which E3 ligases represent specific functions.^75^ The classical E3 ligases regulating cell cycle include APC/C and SCF containing the substrate recognition proteins such as SKP2, β‐TrCP and FBXW7 (Figure 1 and Table 1).^76^, ^77^

**TABLE 1:** E3 ligases implicated in cell cycle.

E3 ligase	Target	Modification	Function of E3 ligase	Role in cancer	Reference
APC/C^CDC20^	Cyclin A	Degradation	Promote anaphase onset	Oncogene	^49^, ^314^
	Cyclin B	Degradation	Promote anaphase onset		
	Securin	Degradation	Promote sister chromatid separation		
APC/C^CDH1^	CDC20	Degradation	Promote mitotic exit	Tumour suppressor	^85^, ^315^, ^316^, ^317^, ^318^
	Plk1	Degradation	Promote mitotic exit		
	Aurora A/B	Degradation	Promote mitotic exit		
	CDC25A	Degradation	Prevent CDK2 accumulation		
	SKP2	Degradation	Prevent CDK2 accumulation		
SCF^SKP2^	p21, p27, p57	Degradation	Promote G1/S transition	Oncogene	^48^, ^94^, ^97^
	Cyclin E	Degradation	Foster S phase progression		
	c‐MYC	Stabilisation	Cell cycle progression		
SCF^β‐TrCP^	EMI1	Degradation	Activate APC/C	Oncogene /Tumour suppressor	^87^, ^90^, ^91^, ^319^
	CDC25A	Degradation	Prevent CDK2 activation		
	WEE1	Degradation	Promote CDK1 activation		
	FOXO3	Degradation	Promote cell cycle progression		
SCF^FBXW7^	Cyclin E	Degradation	Inhibit cell cycle progression	Tumour suppressor	^95^, ^98^
	c‐MYC	Degradation	Prevent c‐MYC expression Inhibit cell proliferation		
CUL3^RhoBTB3^	Cyclin E	Degradation	Inhibit cell cycle progression	Tumour suppressor	^320^, ^321^
Cul4^CDT2^ Cul4^DCAF11^	p21	Degradation	Promote S phase progression	Oncogene	^101^, ^284^
CuL4^DDB2^	p27	Degradation	Promote cell proliferation	Oncogene	^100^
CUL4^AMBRA1^	Cyclin D	Degradation	Inhibit cell proliferation	Tumour suppressor	^322^
MDM2	p53	Degradation	Promote cell proliferation	Oncogene	^223^

APC/C mainly mediates K11 polyubiquitination and degradation of its substrate. It requires its co‐activators CDC20 and CDH1 to regulate the cell cycle between the M phase and early G1 phase.^51^ APC/C^CDC20^ is activated in the M phase by CDK1‐mediated phosphorylation while APC/C^CDH1^ is simultaneously phosphorylated and inhibited until the late M phase when there is a low mitotic kinase level.^78^, ^79^, ^80^ The activated APC/C^CDC20^ is inhibited by the mitotic checkpoint complex at metaphase. This induces metaphase arrest to ensure the correct attachment of sister chromatids to bipolar spindle before transitioning into anaphase.^50^, ^81^ Upon mitotic checkpoint complex satisfaction, APC/C^CDC20^ catalyses the ubiquitination and proteasomal degradation of securin (a separase inhibitor) to promote chromatid segregation, as well as the degradation of cyclin A and cyclin B to promote anaphase onset.^49^ The degradation of cyclin B weakens CDK1 and abates CDK1‐mediated inhibition of CDH1 during anaphase. This activity coupled with CDC14 phosphatase‐induced dephosphorylation of CDH1 promotes the activation of APC/C^CDH1^.^82^, ^83^ Active APC/C^CDH1^ then mediates the proteasome degradation of CDC20, Polo‐like kinase 1 (Plk1) and Aurora kinases (Aurora A and B) at late mitosis to ensure mitotic exit and the degradation of CDC25A (a phosphatase activator of CDK2) and SKP2 at early G1 to lower CDK accumulation.^84^, ^85^ In late G1 phase, early mitotic inhibitor 1 (EMI1) inhibits APC/C and disrupts APC/C^CDH1^‐mediated degradation of its substrates. Although EMI1 is an F‐box protein, its cell cycle regulating activity is F‐box domain independent, to this end, EMI1 regulates APC/C^CDH1^ by antagonising the APC/C^CDH1^ generation the of K11 polyubiquitin chain.^86^ This inhibitory action of EMI1 continues through the S phase and G2 phase until it is degraded by SCF^β‐TrCP^ to allow cell cycle progression through mitosis.^87^ Additionally, SCF^β‐TrCP^ promotes the degradation of CDC25A to suppress cell cycle progression and degrades WEE1 (CDK1 inhibitor) and transcription factor FOXO3 for cell cycle progression.^88^, ^89^, ^90^, ^91^

SKP2 is activated in the late G1 phase by CDK2, which phosphorylates and protects it from APC/C^CDH1^‐mediated degradation.^92^ Once activated, SCF^SKP2^ facilitates G1/S transition by promoting the ubiquitination and degradation of Kip/Cip members of CDK inhibitors (p21^CIP1^, p27^KIP1^ and p57^KIP2^).^48^, ^93^ During the G1 phase, both SCF^SKP2^ and SCF^FBXW7^ target cyclin E for proteasomal degradation^94^, ^95^, ^96^ and act as E3 ligases of the oncogenic transcription factor c‐MYC but with distinct functions. SCF^SKP2^ stabilises and promotes c‐MYC transcriptional activity, while SCF^FBXW7^ mediates proteasome degradation of c‐MYC.^97^, ^98^

In addition to the classical regulators mentioned above, many other E3 ligases including Cul3 E3 ligase complex, Cul4 E3 ligase complex and the SCF containing FBXW8, FBXO4, FBXO7, FBXO31 and FBXL2 have been identified as cell cycle regulators that directly target cyclins and CDK inhibitors as shown in Figure 1.^99^, ^100^, ^101^ More important also is MDM2, the key E3 ligase targeting p53 for proteasome degradation.^102^

Deregulation of cell cycle‐related E3 ligases has been reported in several cancers. SCF^SKP2^ complex is a positive regulator of cell cycle considered as proto‐oncogene because it targets CDK inhibitors and other tumour suppressors. The oncogenic role of SKP2 reconciles with its overexpression in various human cancers including ovarian adenocarcinoma, breast cancer, lung cancer, colorectal cancer, prostate cancer, leukaemia and squamous cell carcinoma.^103^, ^104^, ^105^, ^106^ Interestingly, deletion of SKP2 has been shown to compensate for anti‐tumour and cell safeguarding deficiency in p53 deleted cancer cells due to elevated p27 levels. Zhao and co‐workers demonstrated this using SKP2 knockout mice and found that loss of p53 and pRB in these mice blocked tumorigenesis due to cell cycle arrest mediated by accumulated p27, suggesting that SKP2 could be a promising target for p53 mutant cancer therapy.^107^ Likewise, APC/C co‐activator CDC20 has been identified as an oncoprotein that is highly expressed in several human cancers.^108^, ^109^, ^110^, ^111^, ^112^ Tumorigenic role of CDC20 is linked to its involvement in diverse cellular pathways where it targets tumour suppressors. CDC20 aberrant expression or the disruption of SAC‐mediated inhibition of CDC20 leads to tumorigenesis due to aneuploidy.^113^ Conversely, APC/C^CDH1^ has been associated with a tumour suppressive role as majority of its substrates including SKP2 are known oncoproteins. APC/C^CDH1^‐mediated regulation of SKP2 turnover in cell cycle significantly contributes to its control of tumorigenesis in cells. Studies have shown that inhibition of CDH1 resulted in high cellular proliferation while an induced overexpression in solid tumours is associated with patients’ survival and low histological tumour grade in solid tumours.^114^, ^115^ Nonetheless, SCF^β‐TrCP^ performs dual roles in the cell cycle, promoting both cell cycle progression and cell cycle arrest. This suggests both oncogenic and tumour‐suppressive roles. Hence, the expression of β‐TrCP in cancers is not clearly defined; however, the expression could be linked to a context‐dependent role. The Cul4 E3 ligase complex is also a key regulator of cell cycle that targets CDK inhibitors p21 and p27 as well as cyclin E for degradation. Aberrant expression of Cul4 has been indicated in breast cancer, lung cancer, ovarian cancer and squamous cell cancer.^116^ Studies show that Cul4 depletion reduces the proliferation of lung squamous cell carcinoma and small cell lung cancer.^117^ Moreover, conditional knockdown of Cul4A in mice reduces sensitivity to UV‐induced skin cancer in a mechanism linked to its regulation of p21.^118^ MDM2 is the primary inhibitor of p53 and is amplified in most cancers, especially p53^WT^ cancer types.^119^, ^120^ FBXW7, on the other hand, is an established tumour suppressor that functions as SCF substrate recognition protein targeting several oncogenic proteins including cyclin E and c‐MYC for proteasome degradation. Concomitant with its tumour‐suppressive role, FBXW7 is frequently underexpressed or inactivated in most human cancers.^121^ Taken together, these studies show that E3 ligases are critical regulators of cell cycles, and their cell cycle‐dependent oncogenic function could be considered in the development of therapies that selectively target E3 ligases to induce the cell cycle arrest and corresponding death of cancer cells with considerably lesser toxicity.

### 3.2 Roles of E3 ubiquitin ligases in immune response and inflammation

Evasion of immune response and stimulation of tumour‐promoting inflammation are among the several strategies adopted by cancer cells to sustain proliferation and progression. Host cells through the innate and adaptive immune response, release proinflammatory cytokines that activate macrophages, dendritic cells and natural killer (NK) cells for the destruction of tumour cells and infectious agent that threatens cell immune homeostasis. During immune surveillance, growing cancer cells escape from immune destruction and colonise the infected tissue. They achieve this by promoting the activation of tumour suppressive cells such as the regulatory T cell (Treg), myeloid‐derived suppressor cell and regulatory B cell which suppress anti‐tumour immunity within the tumour microenvironment (TME).^122^, ^123^, ^124^, ^125^ Strikingly, cancer cells promote uncontrollable inflammation which becomes chronic, causing a damaging effect that increases cancer progression.^125^ Evidence of chronic inflammation progression into cancer is exemplified in the study where Mdr2‐knockout mice developed liver inflammation that subsequently progressed to liver cancer.^126^, ^127^ E3 ligases are actively involved in immune response and inflammatory signalling either as promoters or suppressors and could thus be considered novel therapeutic targets for improving anti‐tumour immunity and blocking tumour‐promoting inflammation.

To achieve immune tolerance, cancer cells increase the expression of E3 ligases that promote the activity of Treg and decrease anti‐tumour immunity. Among these E3 ligases, the TRAF6 ligase promotes the activity of Treg within TME by mediating K63‐linked polyubiquitination and activation of FoxP3, a transcription factor for Treg. TRAF6‐deficient Treg cells resist growth of implanted tumours, abolish immune tolerance in cancer cells and enhance anti‐tumour immunity.^128^ The effect of TRAF6 inhibition for immunotherapy was investigated in Hela 1–6 tumour model and the result showed that TRAF6 inhibitors accelerated T cell‐mediated anti‐tumour immunity and blocked Treg infiltration to reduce the Treg tumour population.^129^ Therefore, TRAF6 represents a promising targeting candidate for cancer immunotherapy. Elsewhere, it has also been reported that the E3 ligase ITCH positively regulates Treg by catalysing the monoubiquitination of the transcription factor TIEG1. Monoubiquitination promotes the nuclear translocation of TIEG1 necessary for FoxP3 expression.^58^ Interestingly, ITCH can also mediate IL‐6‐dependent K27‐linked polyubiquitination of TIEG1 which in opposite to monoubiquitination abrogates TIEG1 nuclear translocation thereby preventing FoxP3 expression.^130^ Inhibition of Tregs in this case is found to correlate with an increase in Th17 response and enhance anti‐tumour immunity. Given these contrasting functions of ITCH‐mediated mono‐ and poly‐ubiquitination, targeting ITCH might not be a best option for increasing anti‐tumour immunity but the driving mechanism involved in each case could be considered. Another E3 ligase casitas B‐lineage lymphoma proto‐oncogene‐b (Cbl‐b) has been identified as an important immune checkpoint regulator of CD8^+^ T‐cell and NK cells.^131^, ^132^ Findings from a recent study showed that depletion of Cbl‐b in tumours restores the effector activity of CD8^+^ tumour‐infiltrating lymphocytes and prevents chimeric antigen receptor T‐cell exhaustion.^133^ Given this immune suppressive role, APN401, a small interfering RNA‐based cellular immunotherapy that specifically targets and silences Cbl‐b is currently in clinical studies in patients with advanced solid tumours.^134^

Tumour‐promoting inflammation is mainly regulated via nuclear factor‐kappa B (NF‐κB) signalling. NF‐κB is the key mediator of downstream inflammatory response and is composed of five members (RelA/p65, RelB, c‐Rel, NF‐κB1/p50 and NF‐κB2/p52), from which p52 and p50 are generated from p105 and p100 precursors, respectively.^70^ Both canonical and non‐canonical NF‐κB signalling pathways are regulated by several E3 ligases. For instance, SCF^β‐TrCP^ via a non‐canonical pathway mediates ubiquitination and partial degradation of NF‐κB precursor p100 to generate p52 which then translocates to the nucleus for target gene expression.^135^ In the physiological state, the non‐canonical pathway is inhibited by the E3 ligases cIAP, TRAF2 and TRAF3. These E3 ligases form a destruction complex with NF‐κB‐inducing kinase (NIK) where cIAP conjugates K48‐linked polyubiquitin on NIK to induce its proteasome degradation.^136^, ^137^, ^138^, ^139^, ^140^ When released from the destruction complex, NIK activates downstream IκB kinase alpha (IKKα), which in turn phosphorylates p100 for its partial degradation to p52.^137^, ^141^ Under stimulation, TRAF2 induces cIAP to catalyse proteasome degradation of TRAF3 leading to destruction complex disassembly and consequent release of NIK.^142^, ^143^ Consistent with negative regulation, TRAF2, TRAF3 and cIAPs inactivation mutation has been detected in B‐cell malignancy and their tumour suppressive function is mainly associated with non‐canonical NF‐κB inhibition.^137^, ^144^ ^–^ ^147^

Activation of the canonical NF‐κB signalling pathway promotes nuclear translocation of NF‐κB dimer (RelA‐p50 heterodimer), where they activate target genes. Under normal physiological conditions, NF‐κB inhibitor alpha (IκBα) arrests NF‐κB dimer in the cytoplasm to prevent their nuclear translocation and consequent expression of target genes.^148^ However, with inflammation signalling, IκBα is phosphorylated by IκB kinase (IKK) complex (containing NEMO, IKKα and IKKβ), to promote its ubiquitination and degradation by SCF^β‐TrCP^ E3 ligase.^149^, ^150^, ^151^

**FIGURE 2:**
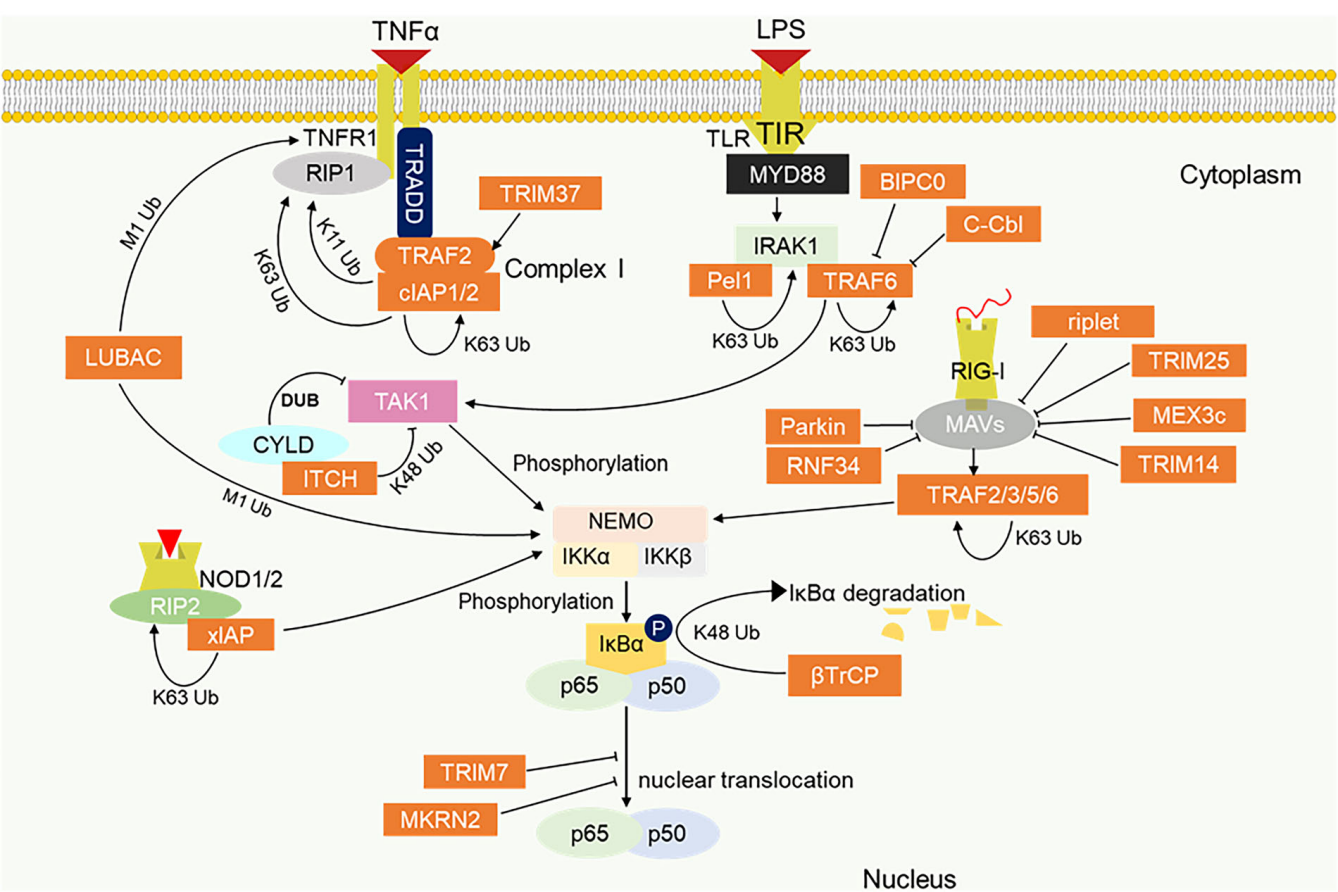
E3 ligase promoting inflammation pathway. Progression of the NF‐κB inflammation pathway via TNFR1 and the pattern recognition receptors: TLR, RIG‐I and NLR, is actively regulated by E3 ligases. K63 and M1 ubiquitin linkages serve as scaffold for the recruitment of downstream activators of NF‐κB. The K48 ubiquitination of IκBα promotes nuclear translocation of p65/p50 for the target gene expression.

Upstream signalling activators of canonical NF‐κB such as tumour necrosis factor receptor 1 (TNFR1) and pattern recognition receptors (PRR) including TLR, RIG‐I‐like receptor (RLR) and NOD‐like receptor (NLR) are regulated by E3 ligases. Upon binding of inflammatory effector ligands (TNFα and cytokines) to their cell surface receptors, TRADD (TNFR‐associated death domain protein) and RIP1 (receptor‐interacting protein 1) are recruited via their death domain to the TNFR1 death domain.^152^, ^153^ TRADD then recruits TRAF2 E3 ligase which further recruits another E3 ligase cIAP to form complex I, undergoing self‐polyubiquitination and K63, K11 and K48 ubiquitination of RIP1 (Figure 2).^154^, ^155^ These polyubiquitin chains act as scaffolds for the recruitment of another ubiquitin ligase LUBAC for M1 linear polyubiquitination of RIP1 and NEMO.^156^, ^157^, ^158^, ^159^, ^160^ TGF‐β activated Kinase 1 (TAK1) is also recruited by the K63 polyubiquitin scaffold, promoting downstream signalling by inducing the phosphorylation and activation of the IKK complex as shown in Figure 2.^161^, ^162^ Key E3 ligases involved in canonical NF‐κB signalling are listed in Table 2. Other E3 ligases regulating NF‐κB signalling include Makorin ring finger 2 (MKRN2) E3 ligase which suppresses NF‐κB signalling via proteasome degradation of p65 and the E3 ligase ITCH which cooperates with deubiquitinating enzyme cylindromatosis tumour suppressor protein (CYLD) to mediate deubiquitination and degradation of TAK1.^163^ Although TRAF2 exhibits anti‐tumour activity via noncanonical NF‐κB, it has also been shown to elicit protooncogenic function by activating canonic NF‐κB signalling.^164^ As TRAF2 is required to interact with cIAP1 or cIAP2 to promote TNFR1‐induced NF‐κB signalling and suppress TNF‐induced cell death, inhibition of TRAF2 or cIAPs is necessary to sensitise tumour cells to TNF‐induced cell death. To this end, several IAP antagonists have been developed for inducing anti‐tumour immunity and have proven effective as a monotherapy or a combination therapy with immune checkpoint blockers such as programmed death 1.^165^, ^166^

**TABLE 2:** Key E3 ligases involved in canonical NF‐κB signalling.

E3 ligase	Ubiquitination mode	Signalling receptor	Function	NF‐κB‐associated role in cancer	References
SCF^β‐TrCP^	K48 polyubiquitination	N/A	Degrade IκBα, promote nuclear translocation of NF‐κB Degrade β‐catenin, promote NF‐κB‐β‐catenin crosstalk	Oncogenic	^135^
TRAF2	K63 polyubiquitination	TNFR1	Polyubiquitinate RIP1, promote recruitment and activation of downstream NF‐κB activators	Oncogenic/tumour suppressor	^323^
cIAPs	K63, K11, K48 polyubiquitination	TNFR1	Polyubiquitinate RIP1, promote recruitment and activation of downstream NF‐κB activators	Oncogenic/tumour suppressor	^231^, ^324^
	K63	NLR	Polyubiquitinate RIP2, promote recruitment and activation of downstream NF‐κB activators	Oncogenic/tumour suppressor	^178^
XIAP	K63	NLR	Polyubiquitinate RIP2, promote recruitment and activation of downstream NF‐κB activators	Oncogenic	^176^, ^325^
LUBAC	M1‐linear polyubiquitination	TNFRI, TLR, RLR	Polyubiquitinate RIP1 and NEMO, recruit IKK complex for activation	Oncogenic	^69^
TRAF6	K63 polyubiquitination	TLR, RLR	Self‐polyubiquitination, promote recruitment and activation of downstream NF‐κB activators	Oncogenic	^168^, ^326^
Pel 1	K63 polyubiquitination	TLR(MD88)	Polyubiquitinate IRAK1 and TAK1 and promote recruitment and activation of downstream NF‐κB activators	Oncogenic/tumour suppressor	^169^, ^327^, ^328^
	K63 polyubiquitination	TRL(TRIF)	Polyubiquitinate RIP1, promote recruitment and activation of downstream NF‐κB activator	Oncogenic/tumour suppressor	
TRIM25,TRIM7 MEX3c, Riplet	K63 polyubiquitination	RLR	Activation of RIG‐I	Not identified TRIM7 is oncogenic	^179^
TRAFs (TRAF2/3/5/6)	K63 polyubiquitination	RLR	Bind MAVs, mediate polyubiquitination for recruitment and activation of IKK complex	Oncogenic/tumour suppressor	^180^, ^181^

TLRs play essential roles in the innate immune system, which include both cell surface and intracellular receptor types. TLRs contain TIR domain through which they recruit adaptor proteins MyD88 (myeloid differentiating factor 88) and TRIF (TIR domain‐containing adaptor‐inducing IFN‐β) for both NF‐κB and/or interferon (IFN) induction.^167^ TRAF6 and Pellino 1 (Pel1) are the key E3 ligases regulating TLR‐induced NF‐κB signalling. They mediate K63 polyubiquitination for induction and activation of TAK1 complex and IKK complex necessary for downstream NF‐κB activation.^168^, ^169^ K63 polyubiquitin is conjugated on TAB2 and TAB3 which complexes with TAK1 (TAK1–TAB1–TAB2 or TAB3 complexes) to aid TRAF6–TAK1 interaction and activation of TAK1.^170^, ^171^ Besides, a ring finger containing E3 ligase BICP0 (bovine herpes virus‐encoded protein, infected cell protein 0), functions as a negative regulator of NF‐κB signalling by mediating K48 polyubiquitination and degradation of TRAF6.^172^ Besides, c‐Cbl has also been reported to inhibit NF‐κB signalling by promoting K48‐linked ubiquitination of TRAF6.^173^ TRAF members particularly TRAF6 are known key regulators of tumour‐promoting inflammation and immune response along TLR–NF‐κB pathway, therefore, small molecule inhibitors of TRAF6‐induced NF‐κB activating inflammation have been identified. Such inhibitor like the TRAF–CD40 inhibitor has been shown to suppress breast cancer metastasis effectively as either a single agent or in combination therapy with conventional chemotherapy.^174^ NLRs and RLRs are cytosolic PRR activated following recognition by bacteria cell walls and cytosolic RNA virus, respectively. These two receptors are master regulators of the innate immune response against pathogens and are considered relevant for inducing immunogenic cell death and anti‐tumour immunity. However, their constitute activation due to dysregulation may elicit proinflammatory signals and chronic inflammation that predisposes to malignancies as such they could be considered a double‐edged sword in inflammation and cancer. NOD1 and NOD2 are the major NLR family that drives NF‐κB signalling. They contain caspase activating and recruiting domain (CARD) for interaction with RIP2.^175^ This complex promotes the recruitment of E3 ligases such as XIAP, cIAPs and LUBAC for recruiting downstream NF‐κB activating cascade.^176^, ^177^, ^178^

Polyubiquitination of RIG‐I by the E3 ligases TRIM25, TRIM14, MEX3c and Riplet (RNF135) is essential for activation and initiation of RIG‐I‐mediated signalling.^179^ RLRs on the other hand include RIG‐I, MDA5 and LGP2 are known to mediate anti‐viral immunity by binding the mitochondria anti‐viral proteins (MAVs). This promotes MAVs interactions with TRAFs (TRAF2/3/5/6) for K63 polyubiquitination and subsequent IKK complex induction.^180^, ^181^ The regulation of NLR and RLR‐driven NF‐κB signalling by E3 ligases has not been clearly described in cancer; however, a few studies have demonstrated the cancer implication of E3 ligase regulation of NOD1/2‐driven NF‐κB signalling. For instance, overexpression of TRIM22 has been shown to decrease the proliferation and migration of endometrial cancer cells as knockdown of TRIM22 was found to accelerate cancer progression via NOD‐NF‐κB pathway.^182^ More so, NOD1 can attenuate *Helicobacter pylori* infection‐induced caudal‐related homeobox 2 (Cdx2) expression and intestinal metaplasia via induction of TRAF3. Since, *H. pylori* infection‐induced Cdx2 expression is dependent on NF‐κB activation, NOD1 induction of TRAF3 inhibits NF‐κB signalling and protects the intestinal cells from precancerous changes.^183^

Wnt/β‐catenin is a significant pathway in carcinogenesis and embryonic development, which has been shown to regulate inflammatory response via cross‐talking with the NF‐κB pathway.^184^, ^185^ SCF^β‐TrCP^ is a key regulator of both Wnt/β‐catenin and NF‐κB signalling pathways. SCF^β‐TrCP^ negatively regulates Wnt/β‐catenin by catalysing K48‐polyubiquitin‐induced proteasome degradation of β‐catenin, subsequently inhibiting its nuclear translocation and target gene expression. It is shown that Wnt/β‐catenin signalling induces high β‐TrCP expression which in turn activates NF‐κB signalling.^186^ Strikingly, an integrated association of β‐TrCP, β‐catenin and NF‐κB is detected in colorectal cancer and is considered important for tumour metastasis and apoptosis inhibition.^187^ In addition, β‐TrCP promotes lymphocytic leukaemia cell proliferation through concomitant activation of NF‐κB and β‐catenin/TCF signalling pathways, suggesting that β‐TrCP–NF‐κB–β‐catenin pathway could be considered a potential target for cancer therapy.^188^

Active Rel/NF‐κB act as a transcription factor for hundreds of genes involved in various biological process including cytokines/chemokines, immunoreceptors, apoptotic regulators, growth factors and transcription factors.^189^ It is reported that increasing NF‐κB signalling is central to driving cancer cell proliferation. As a result, genetic alterations of E3 ligases regulating this pathway have been identified in several human cancers. Oncogenic functions of immunomodulating E3 ligases are mostly attributed to the activation of NF‐κB signalling. For instance, TRAF6 is overexpressed in colon cancer and lung cancer and investigation of its oncogenic function shows the activation of NF‐κB signalling pathway.^190^, ^191^, ^192^ TRAF6‐induced NF‐κB activation promotes multiple myeloma cell adhesion to bone marrow stroma.^193^ Similarly, TRAF2 functions as a NF‐κB activating oncogene that promotes epithelial cancers and skeletal tumour growth in osteotropic breast cancer.^164^, ^194^ Furthermore, mutational activation of BRAF in melanoma promotes β‐TrCP expression and induces NF‐κB activation and melanoma cell growth.^195^ Constitutively, enhanced expression of β‐TrCP in pancreatic carcinoma cells positively correlates with NF‐κB expression and chemoresistance in pancreatic carcinoma.^196^ On the other hand, the U‐box containing E3 ligase CHIP (carboxyl terminus of Hsc70‐interacting protein) as a tumour suppressor weakly expressed in colon cancer and gastric cancer and negatively regulates NF‐κB signalling.^197^, ^198^ CHIP was found to repress the growth of colorectal cancer cells by mediating ubiquitination and degradation of p65 and its expression correlated with TNM stages with the lowest expression in stage 3 and 4 patients.^198^ Further a similar result revealed that TRIM7 E3 ligase negatively regulates NF‐κB signalling by promoting the ubiquitination and proteasomal degradation of p65 in lung cancer.^199^ In this work, Jin and colleagues not only detected low expression levels of TRIM7 in tumours compared to normal cells, but also found that tumour size decreases with stable expression of TRIM7.^199^ With an increasing understanding of the target substrates and mechanisms of action, RNF40 could be considered a target for colorectal cancer treatment. Conversely, TRIM37, another member of the TRIM family, is reported to activate NF‐κB signalling and promote non‐small cell lung cancer aggressiveness due to its K63‐mediated polyubiquitination and activation of TRAF2.^200^ These studies verify the active involvement of E3 ligases in cancer‐promoting inflammatory signalling presenting them as excellent targets for inducing tumour regression via inhibition of NF‐κB tumour‐promotion inflammation.

### 3.3 Evasion of apoptosis by E3 ubiquitin ligases

Cell death is a natural suicidal event for destroying malignant and potentially harmful cells. Of all modes of cell death, programmed cell death (apoptosis) is considered the most significantly inhibited type by cancer cells. This is primarily because it does not elicit adverse effects, thus, diverse therapies targeting apoptotic pathways have been developed for cancer treatment.^201^, ^202^ Table 3 lists significant E3 ubiquitin ligases associated with apoptotic regulation.

**TABLE 3:** E3 ligases of apoptotic signalling.

Intrinsic pathway			
E3 ligase	Activity	Role in apoptosis	References
TRIM17	Degrade Mcl‐1 by ubiquitination	Promote apoptosis	^329^
SCF^β‐TrCP^	Degrade Mcl‐1 by ubiquitination Degrade Bim‐EL by ubiquitination	Context dependent	^219^, ^220^
SCF^Fbxw7^	Degrade Mcl‐1 by ubiquitination	Promote apoptosis	^330^
MULE	Degrade Mcl‐1 by ubiquitination	Promote apoptosis	^331^
APC/^CCDC20^	Degrade Mcl‐1 by ubiquitination	Promote apoptosis	^332^
APC/^CCDH1^	Degrade MOAP1 by ubiquitination	Inhibit apoptosis	^207^
TRIM39	Prevent APC/^CCDH1^ degradation	Promote apoptosis	^207^
XIAP	Inactivate caspase 3/7/9 Ubiquitinate and degrade ccaspase3	Inhibit apoptosis	^209^, ^222^, ^333^
cIAP1/2	Ubiquitinate caspase 3/7, Selfubiquitination, Ubiquitinate Ring IAPs	Inhibit apoptosis	^210^, ^211^, ^212^
AREL1	Ubiquitinate and degrade SMAC	Inhibit apoptosis	^218^

Extrinsic pathway			
cIAPs	Ubiquitinate RIP1, Prevent complex II formation	Inhibit apoptosis	^231^, ^232^
TRAF2	Promote RIP1 ubiquitination and prevent complex II formation	Inhibit apoptosis	^229^, ^334^
	Ubiquitinate and degrade caspase 8	Inhibit apoptosis	^235^, ^236^
LUBAC	Stabilise ubiquitinated RIP1, prevent complex II formation	Inhibit apoptosis	^233^
ITCH	Degrade FLIP	Promote apoptosis	^234^
TRIM9	Upregulate BCL‐2, downregulate caspase 7/9, decrease activity of caspase 3	Inhibit apoptosis	^246^, ^335^
SIAH2 and POSH	Downregulate caspase 8	Inhibit apoptosis	^237^
MKRN1	Degrade FADD	Inhibit apoptosis	^238^

#### 3.3.1 Intrinsic apoptotic pathway

Apoptosis can be propagated through two pathways: intrinsic pathway and extrinsic pathway. The intrinsic pathway is mediated from mitochondria by intrinsic stimuli arising from cellular stress and DNA damage that induce mitochondrial outer membrane permeabilisation (MOMP). There are two regulators of MOMP, the anti‐apoptotic Bcl‐2 members including Bcl‐2, Mcl‐1, Bcl‐XL and Bcl‐w antagonise the formation of MOMP and the pro‐apoptotic Bcl‐2 members (Bax, Bad, Bak, Bid, Noxa, Puma and Bim) which prompt MOMP to release downstream signalling caspase activating proteins, cytochrome *c* and SMAC (Second mitochondria‐derived activator of caspases) for apoptosis.^203^, ^204^ Cytochrome *c* recruits an apoptotic peptidase activating factor‐1 to form an apoptosome complex responsible for activating caspase 9 and downstream effectors, caspases 3 and caspases 7 for apoptosis.^205^ Interestingly, E3 ligases play a critical regulatory role in intrinsic pathways. For instance, Mcl‐1 is found to be polyubiquitinated and degraded by the E3 ligases TRIM17, MULE, SCF^β‐TrCP^, SCF^FBXW7^ and APC/C^CDC20^.^206^ Likewise, TRIM39 E3 ligase inhibits APC/C^CDH1^‐mediated degradation of MOAP1 (Modulator of Apoptosis 1), a tumour suppressor that activates the pro‐apoptotic Bax protein.^207^ These E3 ligases function in the promotion of apoptosis.

In contrast, the IAP family proteins, most importantly cIAPs and XIAP are notable apoptosis inhibitors. XIAPs via their BIR3 domain inhibit apoptosis by binding and inactivating caspases and their BIR2 domain inhibit the catalytic activity of caspases 7 and 3.^208^, ^209^ Utilising E3 ligase activity in apoptosis inhibition, cIAPs mediate ubiquitination of caspase 3/7, self‐ubiquitination and the ubiquitination of other ring finger IAPs,^210^, ^211^, ^212^ while XIAP mediates the ubiquitination and degradation of caspase 3. Unlike XIAP, cIAPs binding to caspases do not inhibit caspase apoptotic function,^213^ therefore, the anti‐apoptotic function of cIAPs could be explained in part by their interaction with TRAFs to activate NF‐κB signalling and prevent TNFα‐induced apoptosis, ubiquitin ligase activity and/or neutralisation of IAP antagonist.^214^, ^215^ SMAC/DIABLO, a natural IAP antagonist from permeabilised mitochondria contains an amino‐terminal tetrapeptide motif with which it binds the BIR domain of IAPs disrupting their anti‐apoptotic function.^216^ SMAC analogues (SMAC mimetics) have currently been developed for promoting the apoptosis of cancer cells.^217^ The SMAC mimetics were originally thought to bind XIAP but subsequent studies reveal that they primarily target cIAPs inducing their autoubiquitination and proteasome degradation and TNFα‐dependent apoptosis, suggesting that cIAPs stabilise the binding and inhibition of caspases by XIAP.^138^, ^139^ Another anti‐apoptotic E3 ligase AREL1 (Apoptosis Resistance E3 Ubiquitin Ligase 1) in turn regulates SMAC by catalysing the ubiquitination and degradation of cytosolic SMAC.^218^ SCF^β‐TrCP^ functions as an anti‐apoptotic E3 ligase promoting the ubiquitination and degradation of pro‐apoptosis protein, Bim‐EL, following Rsk1/2‐induced Bim‐EL phosphorylation. Therefore, inhibition of either β‐TrCP or Rsk1/2 facilitates apoptosis in non‐small cell lung cancer cells.^219^ On the contrary, SCF^β‐TrCP^ also functions as an apoptosis promoter by mediating the degradation of Mcl‐1 following Mcl‐1 phosphorylation by GSK3.^220^ This promiscuous nature of SCF^β‐TrCP^, similar to what was found in cell cycle regulation indicates that the function of SCF^β‐TrCP^ in cancer varies with tissue type. The situation of unrepaired DNA damage triggers p53 to activate the expression of pro‐apoptotic BH3‐only proteins for intrinsic apoptotic signalling.^221^, ^222^ P53‐mediated apoptosis is inhibited by the E3 ligase MDM2 which catalyses the polyubiquitination and degradation of p53 as well as monoubiquitination resulting in cytoplasmic translocation of p53.^223^

#### 3.3.2 Extrinsic apoptotic pathway

**FIGURE 3:**
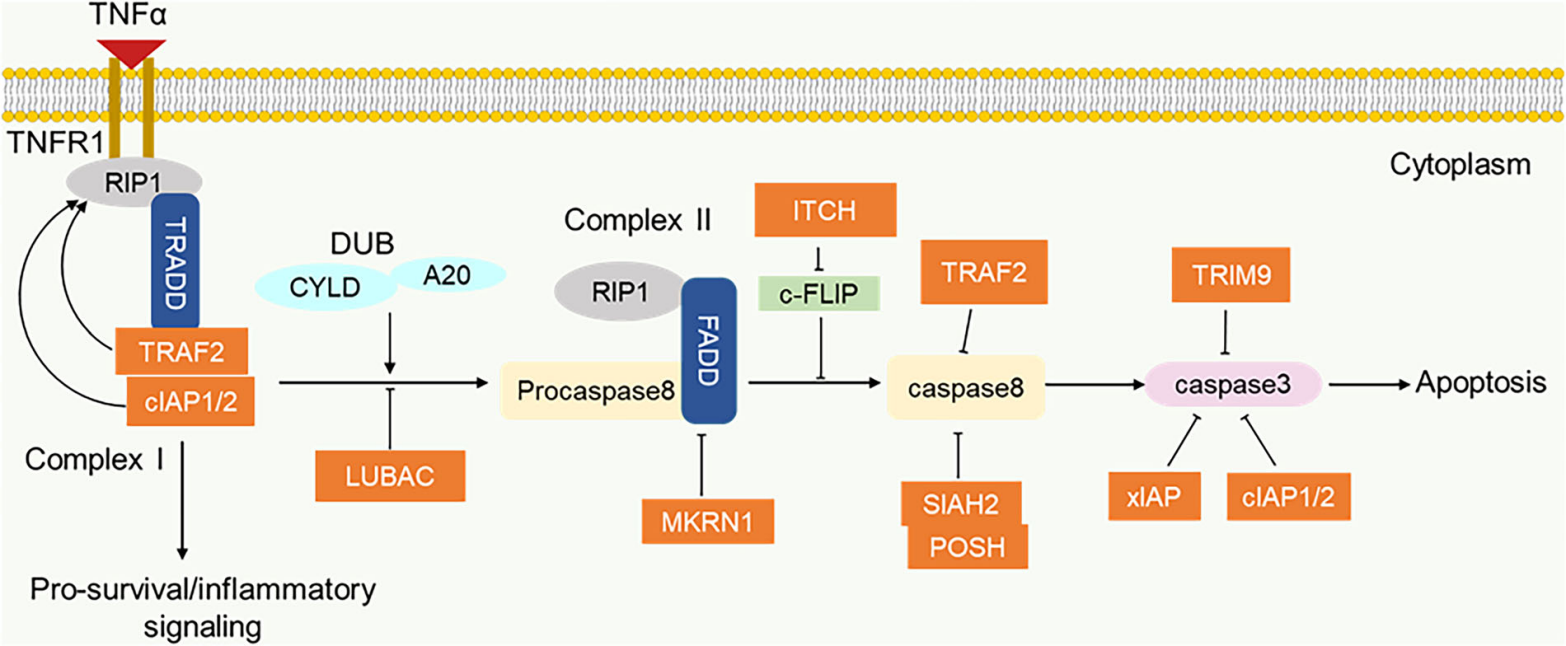
E3 ligases in the regulation of pro‐survival/apoptosis switch. The E3 ligases TRAF2 and cIAP mediate ubiquitination in favour of pro‐survival complex I. The presence of deubiquitinases (CYLD and A20) promote the switch from pro‐survival complex I to apoptotic complex II. LUBAC stabilises the ubiquitin chain in complex I and prevents complex II formation. Several E3 ligases function as negative regulators of apoptotic caspases.

TNFR family containing death receptors (DR) interact with effector ligands such as Fas ligand (Fasl), TNFα and TNF‐related apoptosis‐inducing ligand (TRAIL) and recruit death domain‐containing adaptor molecules (TRADD and FADD) to effectuate intracellular signalling that led to cell death.^224^ Unlike other ligands which trigger only death response, TNFα associates with the receptor TNFR1 and induces either pro‐survival/inflammatory signalling via complex I or apoptotic signalling via complex II.^225^, ^226^ E3 ligases are important regulators of TNFα–TNFR1‐mediated switch from pro‐survival/inflammatory signalling to apoptosis signalling. To induce apoptosis signalling under TNFα stimulation, TNFR1 adaptor TRADD recruits FADD which in turn recruits pro‐caspase 8 to form complex II for activation of downstream apoptosis effector.^227^ TRADD recruitment of the E3 ligases TRAF2 and cIAP on the other hand inhibits apoptosis and promotes complex I formation for pro‐survival/inflammatory signalling.^153^, ^155^ RIP1 can also recruit FADD to form another complex II that comprises RIP1, FADD and caspase 8, so RIP1 interplays between pro‐survival/inflammation and apoptosis signalling.^228^, ^229^, ^230^ Ubiquitination of RIP1 by cIAPs and TRAF2 inhibits RIP1 induction of apoptotic signalling and favours pro‐survival signalling.^229^, ^231^, ^232^ In the absence of cIAP and TRAF2 or in deubiquitinated form, RIP1 dissociated from membrane‐bound complex I to recruit FADD for the formation of apoptotic complex II signalling (Figure 3).^224^ LUBAC is another E3 ligase that impedes RIP1‐dependent apoptosis signalling by mediating the linear ubiquitination of RIP1. This ubiquitination is necessary for recruiting a hybrid protein A20 that blocks CYLD from deubiquitinating RIP1thereby stabilising RIP1 in pro‐survival response.^233^ Cellular FLICE inhibitory protein (c‐FLIP) which interferes with pro‐caspase 8 activation in complex II is inhibited by ITCH that degrades c‐FLIP to promote apoptosis.^234^ In addition, the apoptotic inhibitor, TRAF2 promotes polyubiquitination and degradation of caspase 8 and this contributes to insensitivity to TRAIL ligand‐induced apoptosis in gastric cancer.^235^, ^236^ Likewise, two E3 ligases, Siah2 (Seven in absentia homolog) and POSH (plenty of SH3s), interact together to abrogate TRAIL or FAS ligand‐induced apoptosis by inhibiting downstream caspase 8 activity.^237^ The stability of FADD is impaired in the presence of the E3 ligase MKRN1 which promotes FADD proteasomal degradation.^238^

The expression levels of E3 ligases that negatively affect apoptosis are high in most cancers. An example is seen in XIAP, which is abundantly expressed in anaplastic thyroid carcinoma (ATC). Most notably, this overexpression is associated with proliferation, migration, invasion and chemoresistance in ATC cells.^239^ Additionally, XIAP overexpression has also been reported in breast cancer, renal cancer, colon cancer and leukaemia and correlated with poor overall survival, thus, positioning it as a potential therapeutic for cancer treatment.^240^, ^241^, ^242^, ^243^, ^244^ In a related study, overexpression of TRIM9 is opined to correlate with the upregulation of Bcl‐2 and downregulation of caspases 9/7 leading to apoptosis inhibition in lung cancer.^245^ Similarly, Yang et al.^246^ reported a correlation between TRIM9 overexpression and decreased caspase 3 activity in uterine leiomyoma. TRAF2 shows high expression in prostate cancer cells which is associated with inhibition of TRAIL‐induced apoptosis. In vitro study in prostate DU‐145 cells shows that TRAF2 affects caspase‐8, cFLIP and SIRT1 expression.^247^ Altogether, E3 ligases represent an attractive target that could be considered in clinical application for promoting apoptosis in tumour cells.

## 4 TARGETING E3 UBIQUITIN LIGASE IN THE CANCER HALLMARK PATHWAY

Since the success of the proteasome inhibitor Bortezomib in treating multiple myeloma, there has been a growing attempt towards targeting the ubiquitin–proteasome system for cancer therapy.^248^, ^249^ The new generation therapy is geared toward targeting ubiquitin pathway enzymes (E1, E2 and E3) which act on selected substrates unlike the proteasome inhibitors with broader effects and more associated side effects.^250^ E3 ligases have attracted more attention owing to their specificity, as a result, an increasing number of small molecules targeting E3 ligases have been developed and are currently under clinical trials.^251^ Small molecules targeting E3 ligases of the cell cycle, apoptosis and NF‐κB signalling pathway are elaborated in Table 4 and discussed herein.

**TABLE 4:** Small molecules targeting E3 ligases in cancer hallmark pathways.

Small compound	Targeted E3 ligase	Mode of action	Targeted cancer conditions	Clinical trial phase	References
Nutlin	MDM2	Bind p53 binding pocket in MDM2 and competitively inhibit MDM2–p53 interaction	Osteosarcoma xenograft	N/A	^254^
RG7112	MDM2	Bind MDM2 and inhibit MDM2–p53 interaction.	Leukaemia, advanced solid tumours, liposarcoma	Phase I	^256^, ^258^, ^336^
RG7388 (Nutlin derivative)	MDM2	Bind MDM2 and inhibit MDM2–p53 interaction.	AML, solid tumours, neuroblastoma, breast cancer	Phase I/II (Phase III terminated)	^259^, ^337^ NCT02545283
AMG‐232	MDM2	Bind MDM2 with high potency and inhibit MDM2–p53 interaction	AML, advanced solid tumours, glioblastoma metastatic melanoma, multiple myeloma	Phase I	^261^, ^263^
RITA	MDM2	Specifically bind p53 and induce a conformational change that inhibits MDM2–p53 interaction	Fibro sarcoma and lymphoma cell lines and cervical carcinoma xenograft	N/A	^265^, ^266^
SAR405838 (MI‐773‐01)	MDM2	Bind MDM2 with high affinity and inhibit MDM2–p53 interaction	Malignant neoplasm, lymphoma advanced solid tumours	Phase I	^268^
APG‐115 (AA‐115)	MDM2	Bind MDM2 blocking its inhibitory effect on p53	Advanced solid tumours, lymphoma, advanced liposarcoma, AML T‐prolymphocytic leukaemia	Phase I/II	^338^ NCT04358393 NCT04496349
Milademetan (DS‐3032b)	MDM2	Disrupts MDM2–p53 interaction	Advanced solid tumours, lymphomas, AML, dedifferentiated liposarcoma	Phase I/II/III	^339^, ^340^ NCT0501237 NCT04979442
CGM097	MDM2	Bind MDM2 inhibiting MDM2–p53 interaction	Advanced solid tumours	Phase I	NCT01760525
Siremadlin (HDM201)	MDM2	Bind MDM2 preventing MDM2–p53 interaction	Liposarcoma, AML, Advanced/metastatic soft tissue sarcoma, colorectal cancer	Phase I/II	^341^ NCT05180695 NCT03714958
BI 907828	MDM2	Disrupts MDM2–p53 interaction	Advanced solid tumours, glioblastoma, pancreatic neoplasm dedifferentiated liposarcoma	Phase I/II	^342^ NCT05376800 NCT05512377
MK‐8242	MDM2	Bind to MDM2 and prevent HDM2‐p53 interaction	Solid tumours, AML	Phase I (terminate)	^343^ NCT01463696
Compound A	SKP2	Prevent SKP2 association in SCF complex	Multiple myeloma cell lines	N/A	^269^
Compound #25 (C25)	SKP2	Prevent SKP2 interaction with adaptor SKP1 and inhibit E3ligase activity of SKP2	Liver, lungs, prostate and osteosarcoma cell lines	N/A	^270^
DT204	SKP2	Prevent SKP2 interaction with Cullin1 and Commd1	Myeloma tumours (murine model	N/A	^271^
C series compound (C1, C2, C16, C20)	SKP2	Inhibit Cks1 activity to destabilise SKP2‐p27 interaction	Melanoma cell lines	N/A	^272^
Curcumin	SKP2	Downregulate SKP2 expression	Prostate cancer, pancreatic cancer	Phase I/II/III	^274^, ^275^, ^276^
Dioscin	SKP2	Promote SKP2‐CDH1 interaction necessary for CDH1‐medaited degradation of SKP2	Colorectal cancer cell lines	N/A	^277^
Oridonin	FBXW7	Fbxw7 agonist, promotes degradation of c‐MYC	Leukaemia and lymphoma cell lines	N/A	^278^
Pevonedistat (MLN4924)	CRLs	Inhibit NAE to prevent cullin ring neddylation	Advanced solid tumours, AML, MDS, lymphoma	Phase I/II/III	^282^, ^283^, ^344^
NSC1892	CRL4^DCAF4^	Disrupt Cul4A/B‐DDB1 interaction and prevent CRL4^DCAF4^ formation. Lead to accumulation of ST7, p21 and p27	Colorectal cancer cell lines	N/A	^284^
Pro‐Tame	APC/C	Bind APC/C and inhibit IR tail dependent recruitment of CDC20 and CDH1	NA	N/A	^285^
Apcin	APC/CCDC20	Bind the D‐box binding site of CDC20 and prevent recruitment of APC/CDC20 substrate	NA	N/A	^286^
GS143	β‐TrCP	Inhibit β‐TrCP ubiquitination of IKBα	NA	N/A	^287^
C25‐140	TRAF6	Inhibit TRAF6‐Ubc13 interaction to reduce TRAF6 E3 ligase activity	A study conducted in Autoimmune condition	N/A	^288^
Gliotoxin	LUBAC	Bind the RBR domain of HOIP and inhibit linear ubiquitin chain formation	NA	N/A	^290^
Compound^5^	LUBAC	Bind active cysteine of HOIP and inhibit thioester bond formation	NA	N/A	^291^
HOIPINs	LUBAC	Bind active Cys885 and other associated residue of HOIP and inhibit linear ubiquitin chain formation	NA	N/A	^292^
SMAC mimetic: AT‐406 (DEBIO1143)	IAPs	Bind XIAP and cIAPs to induce cIAP1 degradation and caspase activation	Solid tumours, AML, lymphoma, squamous cell carcinoma	Phase I/II/III	^294^, ^295^, ^345^
SMAC mimetic: GDC‐0152 (Compound 1)	IAPs	Bind BIR3 domains of XIAP and CIAP and BIR domain of ML‐IAP and induce caspase 3/7 activation and degradation of cIAP	Solid cancers	Phase I (Terminated)	^346^ NCT00977067
SMAC mimetic: LCL161	CIAPs	Bind the BIR3 domain of cIAPs and induce their autoubiquitination and degradation	Solid tumours, multiple myeloma, breast cancers, small cell lung cancer	Phase I/II	^347^, ^348^
SMAC mimetic: Birinapant (TL327711)	CIAPs	Bind BIR3 domain of cIAPs to induce their autoubiquitination and degradation	Solid tumours, MDS, ovarian cancers, Neck squamous cell carcinoma	Phase I/II	^296^, ^349^, ^350^
PRT4165	PRC1	Inhibit PRC1‐induced H2A and H2AX ubiquitination	NA	N/A	^298^
GW‐516	PRC1	Catalytically inhibits RNF2 component of PRC1	Prostate cancer cell lines	N/A	^351^
RB‐3	PRC1	Binds RING1B/BMI1 complex and induces conformational changes that disrupt their interaction with nucleosomes	Leukaemia cell lines	N/A	^352^

### 4.1 Small molecules targeting cell cycle E3 ligases

To promote cell cycle arrest and inhibit cellular proliferation in cancer, several small molecule compounds have been developed to target cell cycle E3 ligases such as MDM2, SKP2, FBXW7, APC/C and Cullin4.

#### 4.1.1 MDM2

MDM2 is implicated in both cell cycle and apoptosis due to the broad functions of its substrate, p53, in regulating diverse cellular processes including cell cycle, apoptosis, senescence and DNA repair.^252^ A critical study of the structural basis of MDM2–p53 interaction revealed that the N terminal transactivation domain with conserved amino acids Phe19, Trp23 and Leu26 in p53 is required for interaction with the hydrophobic pocket of MDM2.^253^ This finding prompted the development of small compounds that mimic and bind MDM2 with high affinity. The cis‐imidazole‐containing compound, Nutlin is the first potent MDM2 inhibitor which antagonises MDM2–p53 interaction by acting as a competitive inhibitor that binds to the p53 binding pocket on MDM2.^254^ Nutlin‐3, a derivative of Nutlin, was subsequently developed and it is shown to exhibit anti‐tumour efficacy against tumour cell lines harbouring wild‐type p53.^255^ Accordingly, Nutlin‐3 activates p53‐dependent cell cycle arrest but shows reduced apoptosis induction ability.^255^ Further optimisation of Nutlin‐3 led to the development of RG7112, the first classical MDM2 inhibitor to enter clinical trial. RG7112 effectively induced cell cycle arrest and apoptosis in wild‐type p53 harbouring cancer cells especially solid tumours.^256^

In its phase I clinical trial, RG7112 produced a moderate response but with associated adverse side effects like neutropenia and thrombocytopenia in about half of the patients.^257^, ^258^ For this reason, an improved second‐generation analogue of Nutlin, Idasanutlin (RG7388), was developed and entered clinical trial with results that prove it to be more potent, tolerable and selective than RG7112.^259^, ^260^ However, the phase III clinical trial assessing the overall survival of Idasanutlin as a combination therapy with Cytarabine in patients with relapsed acute myeloid leukaemia (AML) in comparison with only cytarabine and placebo, was terminated for futility based on efficacy results at the interim analysis (ClinicalTrials.gov: NCT02545283). Another MDM2–p53 inhibitor AMG‐232 is a piperidinone‐based compound that also binds the p53 binding pocket in MDM2. AMG‐232 binding with MDM2 showed higher potency than p53 due to additional interaction with glycine58 ‘shelf region’ in MDM2 hydrophobic pocket.^261^ In a preclinical study using tumour xenograft model, AMG‐232 effectively inhibited tumour growth and completely repressed MDM2‐amplified SJSA‐1 via cell cycle arrest and apoptosis.^262^ AMG‐232 progressed into a clinical trial showing modest safety and tolerability with similar but milder side effects than previous MDM2 inhibitors.^263^ The Furan‐derived compound RITA (Reactivation of p53 Induction of Tumour Apoptosis) is another inhibitor of MDM2–p53. Unlike other small compound inhibitors, RITA specifically binds to the p53 N‐transactivation domain rather than MDM2 and induces a conformational change that blocks not only MDM2–p53 interaction but also p53 interaction with other E3 ligase inhibitors.^264^, ^265^, ^266^ RITA is reported to accelerate p53‐dependent apoptosis and inhibit the growth of cancer cells that retain both wild‐type and mutant p53.^265^ Additional important MDM2 inhibitors are the members of the Spiro‐oxindoles class which include the MI series of compounds that mimics p53 binding residue and binds p53 binding pocket in MDM2 to inhibit tumour growth.^267^ Among the M1 series, MI‐773‐01 (SAR405838) has very high binding affinity and specificity for MDM2 and has progressed into clinical trials in patient with solid tumour.^268^ Other important MDM2–p53 inhibitors in clinical studies have been listed in Table 4.

#### 4.1.2 SKP2

Given that SKP2 substrates including p27 and p21 are popular cell cycle inhibitors, small molecule inhibitors of SKP2 inhibit cell growth by inducing cell cycle arrest and cell death. Compound A prevents SKP2 from associating with its SCF complex, leading to G1/S cell cycle arrest and cell death.^269^ Similarly, the small compound inhibitor, Compound #25 (C25) prevents SKP2 interaction with adaptor molecule SKP1 and inhibits E3 ligase activity of SKP2 thereby promoting cell cycle arrest and cell death.^270^ Another SKP2 antagonist of this type is DT204, which overcomes Bortezomib resistance in multiple myeloma by reducing the interaction of SKP2 with Cullin1 and Commd1 adaptor thereby disrupting SCF^SKP2^ complex.^271^ Wu and colleagues identified the C‐series compounds (C1, C2, C16 and C20), which are SKP2 inhibitors that prevent SKP2‐mediated ubiquitination of p27. The C‐series compounds target and inhibit the activities of Cks1, a SKP2‐p27 interacting bridge that promotes SKP2 ubiquitination of p27.^272^ Additionally, the naturally occurring compounds, Curcumin, lycopene and quercetin exhibit anti‐cancer effects by down‐regulating SKP2 expression leading to cell cycle arrest.^273^ Curcumin derived from curcuma longa rhizome (Turmeric) has passed human phase I and phase II clinical trials for cancer and its high safety and no dose‐limiting toxicity enabled progression to phase III clinical in patients with metastatic colon cancer.^274^, ^275^, ^276^ Another natural plant‐derived steroid saponin, dioscin, has been shown to promote CDH1‐mediated polyubiquitination and degradation of SKP2 and a significant reduction in the cell growth of colorectal cancer.^277^

#### 4.1.3 FBXW7 and CRL

FBXW7 as a tumour suppressor is mutated or inhibited in human cancers with high frequency. Oridonin, a naturally occurring compound, is employed as an FBXW7 agonist to promote proteasome degradation of c‐MYC.^278^

CRLs are inhibited by the pevonedistat (MLN4924), a small compound that prevents Cullin neddylation. In the preclinical study using mice model, pevonedistat selectively and efficiently suppressed the growth of human tumour xenograft which facilitated their clinical evaluation.^279^, ^280^ The result of the phase I dose‐escalation and pharmacodynamics study in patients with advanced solid tumours showed that pevonedistat is well tolerated and functions effectively as an NEDD8‐activating enzyme inhibitor.^281^ The phase II and III studies evaluated pevonedistat as a combination therapy with azacitidine in comparison with azacitidine alone in patients with MDS and AML. Here, pevonedistat and azacitidine combination treatment produced a better outcome in phase II study than azacitidine single agent; however, the phase III study failed to meet the prespecified primary endpoint as there was no significant difference between the outcome of the combined therapy and monotherapy.^282^, ^283^ A Cullin scaffold–adaptor interaction is also employed as a target region, by small molecule inhibitor small molecule NSC1892 which specifically disrupts CUL4 interaction with the DDB1 adaptor molecule.^284^

#### 4.1.4 APC/C

The role of APC/C in mitotic progression makes it an important target for inducing mitotic arrest and subsequent tumour death. APC/C co‐activators, CDC20 and CDH1, contain a C‐terminal IR tail and N‐terminal C‐box motif for recruitment to the APC/C complex.^50^ To inhibit APC/C activity, Pro‐Tame (Pro drug of Tosyl‐l‐Arginine Methyl Ester), a small compound that induces spindle checkpoint‐dependent mitotic arrest in human cells, binds APC/C to inhibit IR tail‐dependent recruitment of CDC20 and CDH1.^285^ Another small molecule inhibitor Apcin binds to the D‐box binding site of CDC20 to competitively inhibit APC^CDC20^ ubiquitination of D‐box containing substrates.^286^

### 4.2 Small molecules targeting E3 ligase along inflammatory NF‐κB pathway and apoptotic pathway

Among the targeted pathways for cancer treatment are the tumour promoting inflammation pathway and apoptotic pathway. The strong involvement of E3 ligases in NF‐κB and apoptotic pathways, present them as an important target for inducing apoptosis and inhibiting inflammation in cancer cells. Hence, small compounds targeting the concerned E3 ligases such as SCF^β‐TrCP^, TRAF6, LUBAC and IAPs have been developed for cancer treatment.

β‐TrCP is targeted by the small molecule GS143 for the inhibition of NF‐κB‐mediated inflammation in cancer. GS143 inhibits β‐TrCP‐mediated ubiquitination of IκBα to prevent activation of NF‐κB.^287^

TRAF6 in association with the E2 ubiquitin‐conjugating enzyme Ubc13 mediates K63 polyubiquitination for NF‐κB inflammation signalling.^35^ The small molecule inhibitor of TRAF6, C25‐140, inhibits TRAF6–Ubc13 interaction to reduce TRAF6 E3 ligase activity.^288^ The interaction of CD40 with TRAFs particularly TRAF6 has been shown to activate inflammatory signalling via the NF‐κB pathway. This finding prompted the identification of a small molecule inhibitor of TRAF6–CD40, 6877002 that binds TRAF6 and blocks TRAF6 interaction with CD40 thereby suppressing NF‐κB inflammation signalling.^174^, ^289^

Most small molecule inhibitors of LUBAC, target HOIP, the catalytic subunit in LUBAC. The small molecule, Gliotoxin, inhibits LUBAC by binding to the Ring‐IBR‐Ring domain of HOIP to prevent linear ubiquitin chain formation.^290^ Johansson et al.^291^ designed α, β‐unsaturated methyl ester‐linked compound (Compound 5) which binds the active cysteine to inhibit HOIP E3‐UB thioester bond formation thereby preventing NF‐κB activation. Another similar LUBAC antagonist, HOIPINs bind active Cys885 and other associated residues in HOIP to suppress the RING–HECT‐hybrid reaction of HOIP, thus hindering LUBAC‐mediated linear ubiquitination.^292^

IAP contains a BIR domain that binds caspases in the cell to inhibit cell apoptosis.^208^ Natural IAP antagonist mimetics (SMAC mimetics) are currently developed and under clinical evaluation in cancer subjects.^217^ Small compounds AT‐406 (DEBIO1143), GDC‐0152, LCL161 and Birinapant (TL32711), are SMAC mimetics under clinical evaluation (Table 4), but the clinical trial for GDC‐0152 was terminated after only one phase I study of safety and pharmacokinetics in patients with advanced malignancy although the reason for the termination is not related to patients’ safety or anti‐tumour activity (ClinicalTrials.gov: NCT00977067).^293^, ^294^, ^295^ NF‐κB inflammation signalling and the extrinsic apoptotic pathway crosstalk between complex I and complex II via TNFR1 signalling, and this point could be considered a potential target for cancer therapy.^226^ For instance, TRAF2 recruits cIAP to complex I and prevents the formation of apoptotic promoting complex II. In this case, Birinapant with a high affinity for the BIR3 domain of cIAP binds cIAPs that are associated with TRAF2 to induce their autoubiquitination and degradation.^296^ By this means, Birinapant abrogates complex I‐induced NF‐κB activation and promotes RIP1‐mediated complex II formation for apoptosis of tumour cells.^296^

Besides the polyubiquitinating E3 ligase, small molecule inhibitors of monoubiquitinating E3 ligase such as PRC1 responsible for H2A monoubiquitination have been developed. The catalytic components of PRC1 such as RING1 A or B/BMI1 complex are associated with several cancers and correlate with poor prognosis, thus PRC1 represents an attractive therapeutic target for cancer. PRT4165 was initially designed as small molecule inhibitor of BMI1/Ring1A‐mediated ubiquitination and degradation of topoisomerase II α (Top2α)/drug complex and was later found to inhibit PRC1‐induced H2A and H2AX ubiquitination at DNA damage site. PRT4165 abrogates DNA double‐strand break repair thereby promoting cell cycle arrest and death of DNA‐damaged cells.^297^, ^298^ Su and co‐workers^299^ designed GW‐516, an inhibitor of H2A ubiquitination that catalytically inhibits the RNF2 component of PRC1 to truncate immunosuppression and prevent metastasis of prostate cancer cells. Recently, Grembecka and Cierpicki's laboratory developed the small molecule RB‐3 that inhibits RING1B/BMI1‐mediated H2A ubiquitination in cancer cells. RB‐3 binds RING1B/BMI1 complex and induces conformational changes that disrupt their interaction with nucleosomes. RB‐3 was also shown to induce differentiation in leukaemia cell lines and AML, establishing that RB‐3 may act as a promising therapeutic compound for leukemic cells.^300^

In summary, these studies are convincing evidence that E3 ligases are credible and attractive targets for cancer therapy. The studies also show that small molecule compounds can be directed at E3 ligases, E3 ligase–substrate interaction point or E3 ligase substrates as in the case of RITA to inhibit E3 ligase‐mediated substrate degradation. With a complete understanding of the E3 ligase mechanism within ubiquitin–proteasome system, more efficient and potent E3 ligase inhibitors will be developed for clinical assessment.

## 5 TARGETED E3 LIGASE SUBSTRATES DEGRADATION BY PROTAC IN CANCER THERAPY

Translational therapeutics based on E3 ligase research have evolved beyond small molecule inhibition to induce E3 ligase for the targeted degradation of disease‐causing proteins. This therapeutic approach is accomplished through a class of targeting protein degraders known as the Proteolysis Targeting Chimera (PROTAC) molecule. PROTAC enables chemical hijacking of the endogenous proteasome system to degrade the protein of interest (POI). Since it was first proposed in 2001,^301^ the PROTAC technology has attracted significant interest from academia and industry due to its demonstrated advantages over small molecule inhibitors. Significant advantages of the PROTAC molecule include its event‐driven pharmacology, reversible catalytic mechanism, selectivity and ability to modulate enzyme–non‐enzyme proteins and transcription factors that are considered non‐digestible by traditional small inhibitor molecules.^302^, ^303^

**FIGURE 4:**
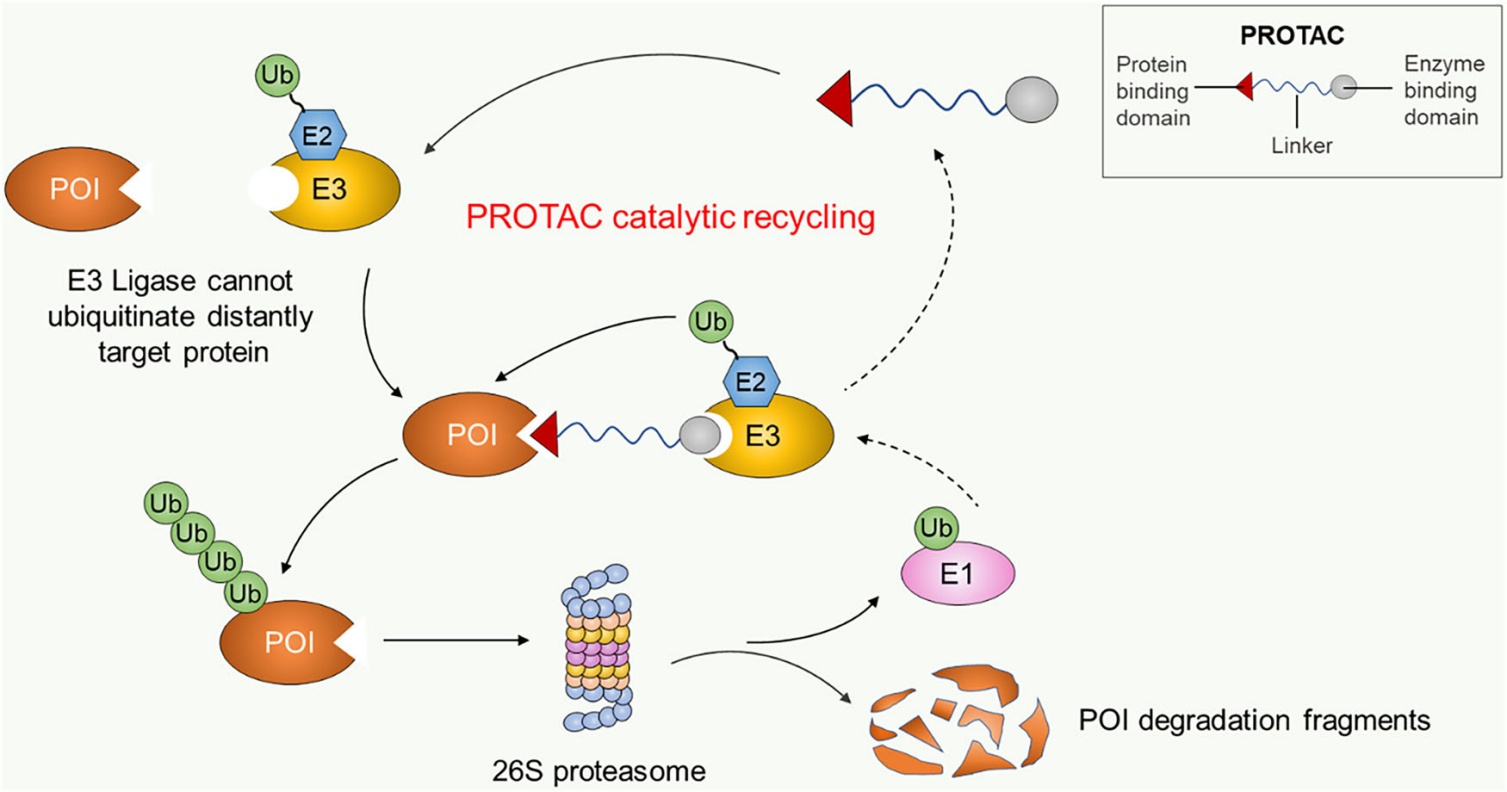
The mechanism of PROTAC‐mediated target protein degradation. PROTACs simultaneously recruit POI and E3 ligase into a ternary complex where ubiquitin is transferred from the E2–E3 complex to POI. Ubiquitinated proteins are attracted to the 26S proteasome system for degradation while PROTAC is recycled to recruit new targets.

PROTACs are heterobifunctional small molecules composed of two ligand warheads (target protein–ligand and E3 ligase ligands) and a linker connecting them. This design facilitates the recruitment of POI and E3 ligase nearby within a ternary complex for target protein ubiquitination and proteasome degradation.^301^, ^304^ The mechanism of PROTAC‐mediated degradation of POI is catalytic and allows for iteration, since after induction of ubiquitination, PROTAC dissociates from the ternary complex to recruit new targets as shown in Figure 4. Unlike occupancy‐driven small molecule inhibitors that modulate protein functions by binding to active sites, PROTACs eliminate target proteins by binding alternative non‐active sites. This modality enhances the pharmacodynamic action of PROTACs due to the need to resynthesise new proteins and limits the potential for mutational drug resistance.^305^, ^306^ While the binding affinity for the target protein is the key determinant of the potency of small molecule inhibitors, the degradation efficiency of PROTACs has other influencing factors such as the cooperativity of the ternary complex, the linker size and the choice of E3 ligase, all of which are highly considerable factors in the design and optimisation of PROTACs for efficiency.^307^, ^308^, ^309^, ^310^, ^311^, ^312^ An excellent review by Rao's group discussed in detail the strategies in the design and optimisation of PROTACs for potency.^313^

## 6 CONCLUSION AND FUTURE PERSPECTIVE

In previous studies, E3 ligases are known to mediate substrate monoubiquitination or polyubiquitination through their substrate recognition function and without doubt play a classic role in the ubiquitination process. However, the mechanism underlying monoubiquitination and polyubiquitination of one substrate by the same E3 ligase remains to be fully understood. For instance, MDM2 catalyses both monoubiquitination and polyubiquitination of p53 with different effects, as such insight into the mechanism that prompts mono‐ versus poly‐ubiquitination for one substrate as well as other ubiquitin chains including branched chains and heterotypic chains would be useful in defining E3 ligase specificity and optimising the design of small molecule E3 ligase‐based therapies.

The specificity of E3 ubiquitin ligases as well as their critical involvement in both normal cell function and cancer hallmark pathways have attracted much attention for possible application in cancer therapy. The understanding of E3 ligase–substrate interaction has facilitated the design of small molecule compounds targeting E3 ligases as inhibitors, modulators or agonists. Oncogenic E3 ligases are overexpressed in most cancers and are targeted by inhibitors while tumour‐suppressing E3 ligases are underexpressed or mutated in some cancer types and are targeted by agonists. Some substrates are targeted for ubiquitination by more than one E3 ligase exemplified in p53 which serves as a substrate for over five E3 ubiquitin ligases. In this case, targeting and inhibiting just one of these E3 ligases might not effectively control tumour progression as other E3 ligases would likely act on the substrate to counteract the effect of the inhibitor. In addition, some E3 ligases play a contrasting function in cell processes as both oncogene and tumour suppressors, therefore, therapies regarding this class of E3 ligases would be more effective at targeting E3 ligase–substrate interaction than targeting the E3 ligases.

The majority of the small molecule compounds targeting E3 ligases are inhibitor compounds that inhibit oncogenic E3 ligases leading to an increased level of their substrates. However, the growing interest in more recent years is towards harnessing E3 ligase machinery for degrading oncogenic proteins. The target protein degradation technology PROTAC has opened a new phase in drug design with its overwhelming advantages over small molecule inhibitors. Although this new technology has seen remarkable clinical success within a few years of development, there are still concerning challenges that need to be addressed and they include structural, kinetics and dynamics understanding of ternary complex, computational approach for studying ternary complex interactions, effective screening for easy identification of ligand, effecting approach for expanding the scope of hijackable E3 ligase and for expanding the library of undruggable targets. To this end, high throughput techniques that address these challenges would aid a complete understanding of the PROTAC‐mediated degradation mechanism for improved PROTAC design and optimisation.

In the past few years, E3 ligase has gained substantial research interest which has fostered a rising concern in E3 ligase‐based therapeutic intervention. Several E3 ligase‐based therapies are under clinical evaluation for cancer treatment and some like the E3 ligase modulators (thalidomide, lenalidomide and pomalidomide) have been approved by FDA in patients with multiple myeloma. The emergence of target protein degraders particularly PROTACs has strikingly increased the number of E3 ligase‐based therapies in clinics and with the increasing successful clinical outcomes, the coming years will have more FDA‐approved E3‐ligase‐based therapies for cancer treatment.

## CONFLICT OF INTEREST STATEMENT

All authors declare no competing interest.
